# Rapid Analysis of Compounds from Piperis Herba and Piperis Kadsurae Caulis and Their Differences Using High-Resolution Liquid–Mass Spectrometry and Molecular Network Binding Antioxidant Activity

**DOI:** 10.3390/molecules29020439

**Published:** 2024-01-16

**Authors:** Dezhi Shi, Nanxi Liao, Hualan Liu, Wufeng Gao, Shaohui Zhong, Chao Zheng, Haijie Chen, Lianlian Xiao, Yubo Zhu, Shiwen Huang, Yunyu Zhang, Yang Hu, Yunfeng Zheng, Jing Ji, Jianming Cheng

**Affiliations:** 1 College of Pharmacy, Nanjing University of Chinese Medicine, Nanjing 210023, China; 20210720@njucm.edu.cn (D.S.); 20210928@njucm.edu.cn (N.L.); 20210891@njucm.edu.cn (H.L.); 20210723@njucm.edu.cn (W.G.); 20220799@njucm.edu.cn (S.Z.); 20210865@njucm.edu.cn (C.Z.); chenhaijie039x@163.com (H.C.); 20210939@njucm.edu.cn (L.X.); 15371723103@163.com (Y.Z.); hshw_66@163.com (S.H.); 20200665@njucm.edu.cn (Y.Z.); huyang@njucm.edu.cn (Y.H.); zyunfeng88@126.com (Y.Z.); 2 Jiangsu Province Engineering Research Center of Classical Prescription, Nanjing 210023, China

**Keywords:** Piperis Herba, Piperis Kadsurae, UPLC-Q-Zeno-TOF-MS/MS, molecular network, antioxidant activity

## Abstract

There is a serious mixing of Piperis Herba and Piperis Kadsurae Caulis in various parts of China due to the similar traits of lianas, and there is a lack of systematic research on the compound and activity evaluation of the two. Likewise, the differences in compounds brought about by the distribution of origin also need to be investigated. In this study, high-resolution liquid–mass spectrometry (UPLC-Q-Zeno-TOF-MS/MS) was used to analyze samples of Piperis Herba from five origins and Piperis Kadsurae Caulis from five origins, with three batches collected from each origin. The compounds were identified based on precise molecular weights, secondary fragments, and an online database combined with node-to-node associations of the molecular network. The *t*-test was used to screen and analyze the differential compounds between the two. Finally, the preliminary evaluation of antioxidant activity of the two herbs was carried out using DPPH and ABTS free radical scavenging assays. The results showed that a total of 72 compounds were identified and deduced in the two Chinese medicines. These compounds included 54 amide alkaloids and 18 other compounds, such as flavonoid glycosides. The amide alkaloids among them were then classified, and the cleavage pathways in positive ion mode were summarized. Based on the *p*-value of the *t*-test, 32 differential compounds were screened out, and it was found that the compounds of Piperis Herba were richer and possessed a broader spectrum of antioxidant activity, thus realizing a multilevel distinction between Piperis Herba and Piperis Kadsurae Caulis. This study provides a preliminary reference for promoting standardization and comprehensive quality research of the resources of Piperis Herba using Piperis Kadsurae Caulis as a reference.

## 1. Introduction

Both Piperis Herba and Piperis Kadsurae Caulis are from Piperaceae, and they are known to expel wind dampness, open the meridians, and relieve paralyzing pain. They are widely used in the treatment of joint pain in the limbs. Modern research has found that Piperis Herba and Piperis Kadsurae Caulis can increase coronary blood flow, which is applied to various diseases, such as coronary heart disease and angina pectoris, and has a certain protective effect on platelet-activating factor hypotension and pulmonary edema [1]. In terms of pain relief, some studies show that Piperis Herba and Piperis Kadsurae Caulis can raise the pain threshold and that they have a good therapeutic effect on inflammatory pain [2]. It has also been found that Piperis Herba and Piperis Kadsurae Caulis have a certain protective effect against oxidative damage in the body, and they have free radical scavenging activity at the same time [3]. The pharmacological activities of Piperis Herba and Piperis Kadsurae Caulis are very extensive and have important application prospects.

However, both herbs are climbers and have very similar traits and functions. This has resulted in a very serious mixing of the two herbs in many regions. Piperis Kadsurae Caulis is listed in the Chinese Pharmacopoeia [4], while Piperis Herba is included in the local pharmacopoeia standards of different regions in China, with varying descriptions. It is still not included in the national standard because of many factors, such as mixed origins and varieties. Although both of them have certain effects of dispelling wind and relieving pain, there are some differences, and the mixing of the two will cause the original rational drug to lose its therapeutic effect [2]. Due to the variety and origin, the study of the two species is still relatively limited, and the compound of the two species has been studied in alkaloids [5], lignans [6] and volatile oils, but no systematic characterization of the two species has yet been found, so the identification of the differential compounds of the two species needs to be urgently resolved.

To further clarify and compare the differences in the compounds of Piperis Herba and Piperis Kadsurae Caulis, a high-resolution liquid–mass spectrometer, ZenoTOF 7600, was selected for rapid scanning and analysis. In previous studies, alkaloids were found to have significant pharmacological activities as the characteristic representative compounds of the two, but due to the similarity of their compounds and the limitation of controls, it is difficult to rapidly and accurately identify the compounds [7]. At present, the molecular network has been widely used in the rapid identification of compounds of natural medicines [8]. Compared to feature-based molecular networks, classical molecular networks retain a richer set of nodes that can provide more information and networking [9]. On the other hand, the molecular network is also able to visualize the different data collected through Cytoscape, forming a pie chart indicating the relative content and thus achieving a preliminary comparison [8]. Markview enables peak extraction, peak comparison and isotope removal for multiple batches of data, and *t*-tests for common peaks for multiple batches to analyze differential compounds [10]. In this experiment, we focused on the antioxidant activity of both [11], choosing the more widely used DPPH and ABTS free radical scavenging assays for a preliminary comparison of the antioxidant activity of Piperis Herba and Piperis Kadsurae Caulis [12]. The above analytical methods were used in order to achieve a differential evaluation of the composition and efficacy of the multi-origin, multi-batch sources of Piperis Herba and Piperis Kadsurae Caulis.

Therefore, the present study allows for the rapid identification of differential compounds of Piperis Herba and Piperis Kadsurae Caulis and the preliminary evaluation of the antioxidant activity of both. The above study is important for accelerating the national standardization of Piperis Herba and rational compounding of the two herbs.

## 2. Results

### 2.1. Optimization of Extraction and Mass Spectrometry Conditions

The results showed that choosing the material–liquid ratio of 1:100 for the extraction of 75% methanol for 30 min allowed for detecting the majority of the substances. Considering that the alkaloid compounds were almost unresponsive in the negative ion mode, the positive ion mode was finally adopted for the collection after comparison. The results of the optimization of the conditions are presented in Supplementary Material S1.

### 2.2. Molecular Network Visualization and Analysis

**Figure 1:**
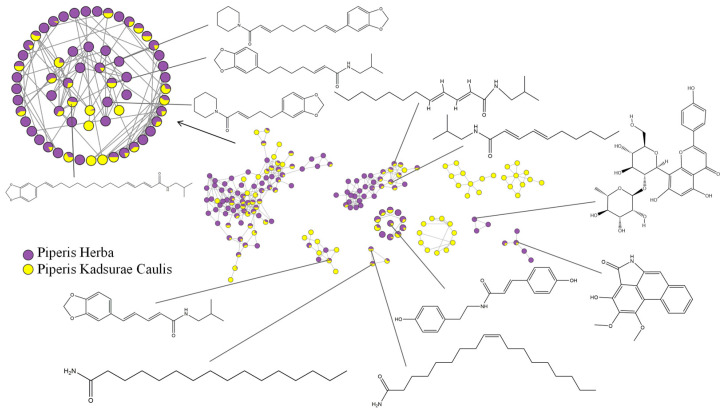
Molecular network visualization diagram in positive ion mode.

The identified substances were subjected to molecular network visualization, and the results are shown in Figure 1. The amide alkaloids, as the main compounds in both Piperis Herba and Piperis Kadsurae Caulis, were clustered into two main networks in the molecular network. The first one was dominated by the amide alkaloids containing piperidinium, isobutyl, and pyrrolidinium, which was characterized by the presence of the 1,3-benzodioxol-5-yl group; the second network was composed of fatty amide alkaloids that dominated the second cluster network; some of the long-chain fatty amides were clustered into a separate network due to their structural similarity to fatty acids; and the long-chain fatty amide alkaloids were clustered into a cluster network. The distribution of compounds in Piperis Herba and Piperis Kadsurae Caulis can be visually compared based on the figure, so the next step is to analyze the compositional differences between Piperis Herba and Piperis Kadsurae Caulis from multiple origins.

In this experiment, UPLC was firstly carried out to investigate the extraction solvents (water, 50% methanol, 75% methanol, and methanol), extraction time (15, 30, and 60 min), material–liquid ratios (1:25, 1:50, 1:100, and 1:200 *w*/*v*), and the gradient of the mobile phase (acetonitrile–water, methanol–water, acetonitrile–0.1% (*v*/*v*) formic acid in water). The results showed that choosing the material–liquid ratio of 1:100 for the extraction of 75% methanol for 30 min allowed for detecting the majority of the substances. Considering that the alkaloid compounds were almost unresponsive in the negative ion mode, the positive ion mode was finally adopted for the collection after comparison.

### 2.3. Identification of the Compounds of Piperis Herba and Piperis Kadsurae Caulis

**Figure 2:**
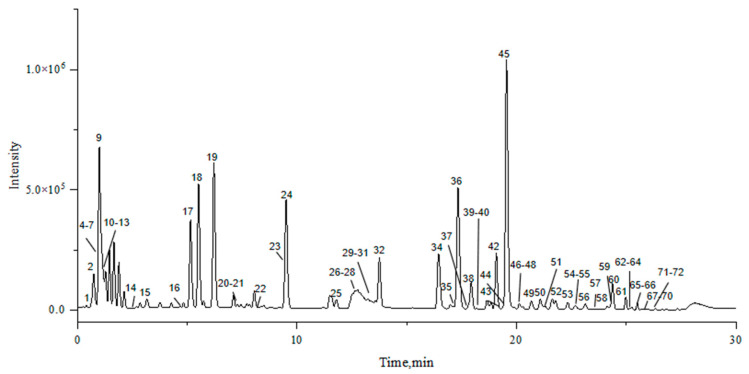
The total ion chromatogram (TIC) of Piperis Herba from Guangxi Guilin in the positive ion mode.

**Figure 3:**
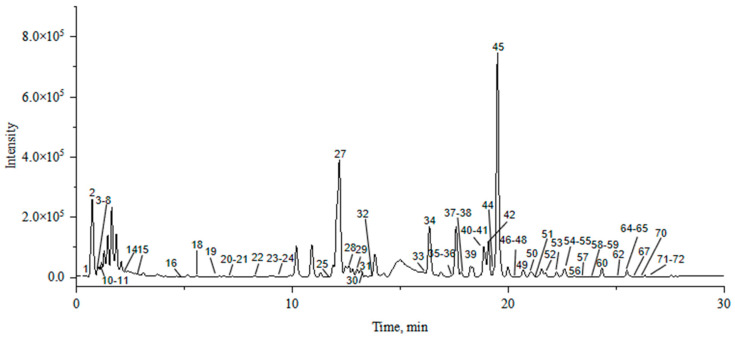
The total ion chromatogram (TIC) of Piperis Kadsurae Caulis from Sichuan Yunlian in the positive ion mode.

The molecular network results were processed using peakview to exclude false-positive results, and the compounds of the samples from Piperis Herba and Piperis Kadsurae Caulis were identified based on the association between nodes and nodes. A total of 72 compounds were identified, including 54 alkaloids and 18 other compounds, such as flavonoid glycosides and volatile oils. The results showed that the compounds identified in Piperis Herba from Guangxi Guilin and Piperis Kadsurae Caulis from Sichuan Yunlian were more representative. Figure 2 shows the total ion chromatogram (TIC) of Piperis Herba from Guangxi Guilin in the positive ion mode, and Figure 3 shows the BPCs of Piperis Kadsurae Caulis from Sichuan Yunlian in the positive ion mode. Detailed information regarding the identified compounds is shown in Table 1, and their corresponding structures are given in Supplementary Material S2.

### 2.4. Identification of the Amide Alkaloids

**Figure 4:**
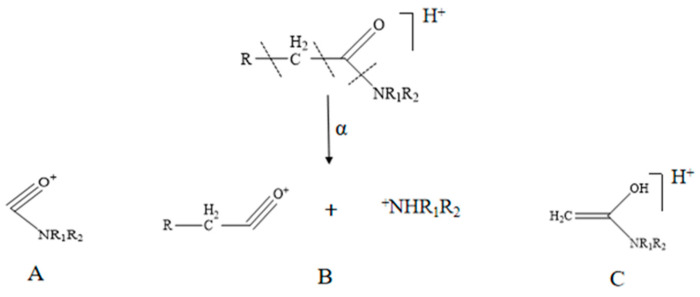
Schematic representation of three cleavage modes of amide alkaloids in positive ion mode. **A** (acyl carbon atom initiated α-cleavage), **B** (acyl nitrogen atom initiated α-cleavage) and **C** (γ-hydrogen rearrangement).

Amide alkaloids are the main compounds and the main active ingredients of both Piperis Herba and Piperis Kadsurae Caulis. Alkaloids are mainly nitrogen-containing organic compounds, and the majority of the 54 alkaloids identified in this experiment were straight-chain amide alkaloids, including 20 isobutyl amide alkaloids, 12 piperidine amide alkaloids, and 9 pyrrolidine amide alkaloids. The cleavage modes of these amide alkaloids were mainly three types of alpha cleavage, which was characterized by the cleavage triggered by oxygen and nitrogen atoms of the acyl group and the subsequent generation of different fragments. Several α-cleavage modes of these amide alkaloids are shown in Figure 4 [30].

**Figure 5:**
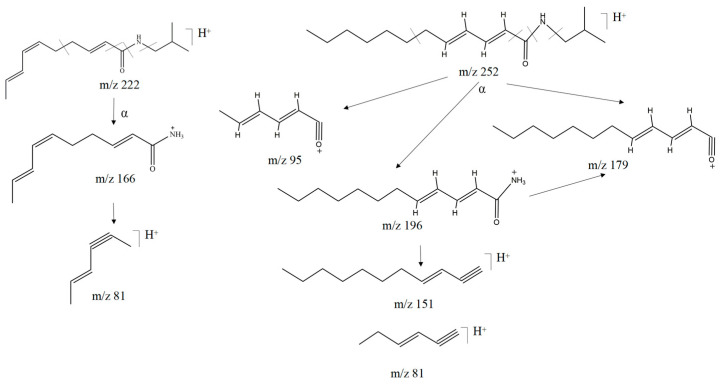
Possible cleavage pathway for compounds **38** and **60** in positive ion mode.

The amide alkaloids of the isobutyl group can be divided into two classes, of which compounds **38**, **45**, **52**, **60**, **64**, **70,** and **72** are fatty amide alkaloids, which are cleaved mainly by class A and B α-cleavage in Figure 4. For example, compound **38** is cleaved by the oxygen and nitrogen atoms in the amide bond, generating a fragment peak with *m*/*z* 166 (C_10_H_16_NO^+^) due to the neutral loss of isobutyl, followed by the α-cleavage of the carbonyl group and the loss of the alkyl fragments, generating a fragment peak with *m/z* 81 (C_6_H_8_^+^). Compound 60 has a similar cleavage pattern, with the neutral loss of either isobutyl or *m/z* 73 (C_4_H_11_N) generating a fragment peak with *m/z* 196 (C_12_H_22_NO^+^) or 179 (C_12_H_19_O^+^), or through the loss of CO followed by alkyl fragmentation to generate a fragment peak with *m*/*z* of 95 (C_6_H_7_O^+^). The cleavage patterns of compounds **38** and **60** are shown specifically in Figure 5, and combined with the above analyses, the identification of the remaining fatty amide alkaloids was completed through the website and based on the literature.

**Figure 6:**
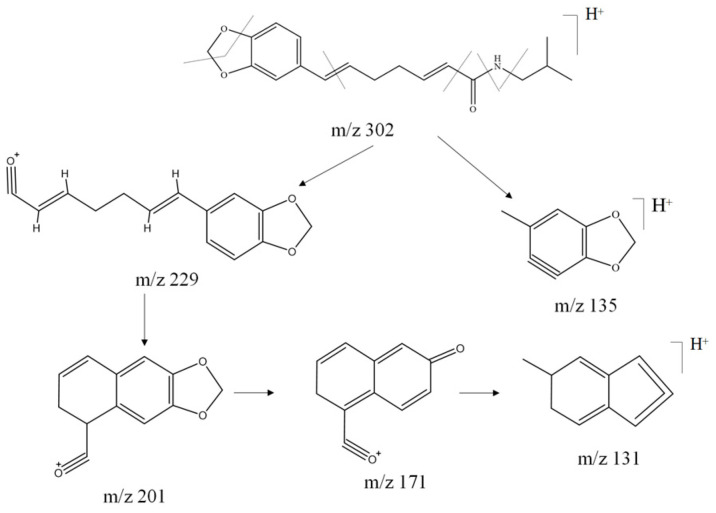
Possible cleavage pathway for compound **34** in positive ion mode.

Compounds **18**, **27**, **34**, **37**, **40**, **46**, **48**, **54**, **58**, **65**, **66**, **68,** and **71** comprise a second class of isobutyl amido alkaloids characterized by the presence of the 1,3-benzodioxol-5-yl group and isobutylamine. These compounds also contain cleavage of the 1, 3-benzodioxol-5-yl group through the loss of CH_2_O to obtain a fragment ion with an *m/z* of 135 (C_8_H_6_O_2_^+^). Compound **34** is mainly cleaved by class A alpha cleavage to obtain a *m/z* of 229 (C_14_H_13_O_3_^+^), and the fragment ions with *m/z* of 201 (C_12_H_9_O_3_^+^), 171 (C_11_H_7_O_2_^+^), and 131 (C_10_H_10_^+^) were obtained through multiple rearrangements of hydrogen, respectively. On the other hand, there is also a structure containing the 1, 3-benzodioxol-5-yl group remaining after class B α-cleavage through loss of CH_2_O to obtain fragment ions with *m/z* of 135 (C_8_H_6_O_2_^+^), and the specific cleavage pattern is shown in Figure 6. Combining the two cleavage patterns, the remaining isobutyl alkaloids can be structurally characterized.

**Figure 7:**
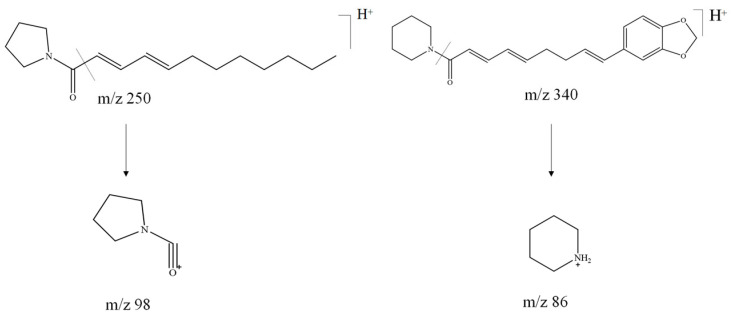
Possible cleavage pathway for compounds **47** and **55** in positive ion mode.

The amide alkaloids of the piperidine class, the most biologically important precursors that include the piperidinium salts, are characterized by cleavage by amide bond class B alpha cleavage to give characteristic fragment ions with *m/z* of 86 Da (C_5_H_12_N^+^), of which compounds **23**, **25**, **29**, **30**, **35**, **36**, **39**, **47**, **50**, **53**, **59**, **61**, **63**, and **69** were identified as piperidine amide alkaloids. The amide alkaloids of the pyrrolidine class are similar to the amide alkaloids of the piperidine class, which include **19**, **21**, **24**, **32**, **51**, **55,** and **56,** and their cleavage is characterized by a characteristic fragment ion with *m*/*z* of 72 (C_4_H_10_N^+^) via an amide bond via class B alpha cleavage, which, however, could not be detected due to the scanning range setting, but it can be identified by class A α cleavage to obtain *m*/*z* of 98 (C_5_H_8_NO^+^). The specific cleavage pathways exemplified by compounds **47** and **55** are indicated in Figure 7. The other piperidine and pyrrolidine alkaloids were resolved in combination with the isobutyl amide alkaloids.

The rest also include one simple amide alkaloid and three dimeric amide alkaloids, which share a similar cleavage pattern to the above alkaloids in the positive ion mode via class B alpha cleavage to produce fragment ions with an *m*/*z* of 105 Da (C_7_H_5_O^+^). Apophilic amide alkaloids undergo rearrangement through the loss of CH3· and CO and then undergo a rearrangement, such as compound **20,** through cleavage to form fragment ions with *m*/*z* of 251 (C_16_H_11_O_3_^+^) and 195 (C_14_H_11_O^+^).

### 2.5. Analysis of the Differences in the Compounds between Piperis Herba and Piperis Kadsurae Caulis

**Figure 8:**
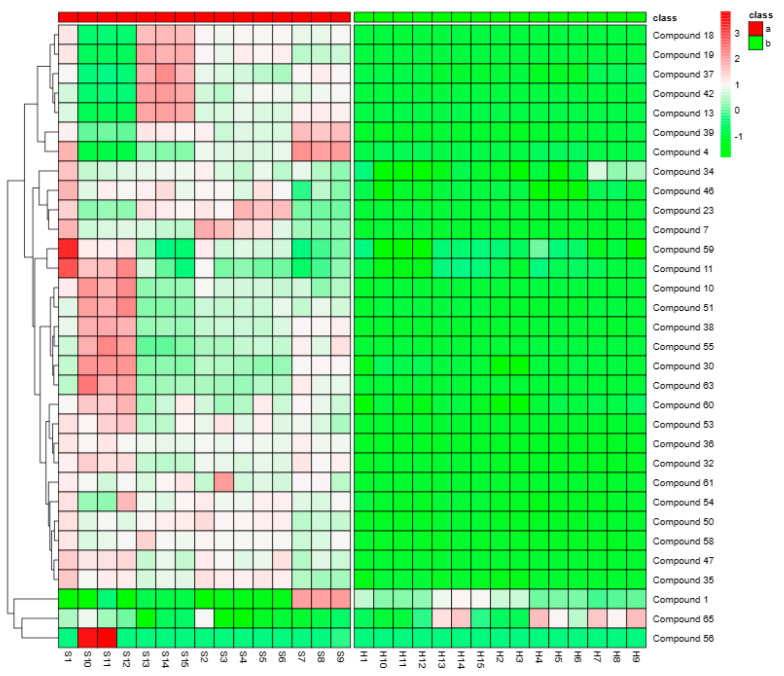
Heatmap of differential compounds of Piperis Herba and Piperis Kadsurae Caulis.

The differential compounds between 15 batches of Piperis Herba and 15 batches of Piperis Kadsurae Caulis were analyzed using a *t*-test, and the compounds with *p* < 0.05 were selected as differential compounds, from which 32 differential compounds were screened out and identified. The peak areas of the differential compounds of each batch were used as their relative contents to draw the clustering heatmap so as to achieve the intuitive description of the differential compounds of multiple batches. Among the 32 differential compounds screened, 29 alkaloids, 2 flavonoid glycosides, and 1 volatile oil compound were identified, except for compound 65, which had a higher relative content in Piperis Herba. The distribution of the differential compounds with amide alkaloids as the main compound was more abundant in Piperis Herba, and the quality of Piperis Kadsurae Caulis was less affected by the difference in origin relative to Piperis Herba. The quality of Piperis Herba from different origins was more varied than that of Piperis Herba from different origins, among which compounds **13**, **18**, **19, 37,** and **42** had higher relative contents in Piperis Herba from Guangxi Yulin, while compounds **4** and **39** were widely distributed in Guangxi Baise. The compound from Yunnan Dali was the richest, and the compound from Guilin, Guangxi was the most abundant. The proportion of vine stems and leaves in them has some correlation. The majority of the three batches of Piperis Herba from Guangxi Guilin collected in this experiment was in the form of vine stems in S1, while a certain proportion of leaves were mixed in S2 and S3, which is possibly one of the reasons for the large differences in Piperis Herba. The results were analyzed and evaluated, and it was found that the relative content of the different compounds in Piperis Herba was higher as a whole, so it was possible to differentiate between Piperis Herba and Piperis Kadsurae Caulis in terms of compounds. It was also found that the quality of Piperis Kadsurae Caulis was relatively more stable and that Piperis Herba was used as vine stems for medicine, while Piperis Herba was used as leafy vine stems for medicine. The differences that existed between the vine stems and the leaves would bring about the difference in the compound, and then the difference in the efficacy of the medicine, which provides ideas for the stability of the quality of the Piperis Herba. The results were analyzed and evaluated, and it was found that the relative contents of the differential compounds were higher in Piperis Herba, thus distinguishing Piperis Herba and Piperis Kadsurae Caulis in terms of compounds. The heatmap of the differential compounds is shown in Figure 8.

Pellitorines are found in high relative amounts in both Chinese medicines, and they have also been reported in the literature to have antipyretic, analgesic, and neuroprotective effects [6]. This suggests that this constituent may make the two Chinese medicines show similar efficacy to some extent, but it is not a differential constituent, indicating that the substance with high relative content may not be the key efficacious constituent of the drugs. The study of the differential components revealed that many of them stayed in preliminary studies. For example, with regard to Pipersintenamide, which has a certain relative content in Piperis Herba, only the structural characteristics of this component were reported, and the evaluation of its activity has not yet been retrieved [23]. For Guineensine, the relative content is higher in Piperis Kadsurae Caulis, and its neuroprotective effect has been reported in the literature [6]. These compounds suggest to some extent the therapeutic effects of our two drugs.

### 2.6. Results of DPPH and ABTS Free Radical Scavenging Experiments in Piperis Herba and Piperis Kadsurae Caulis

**Figure 9:**
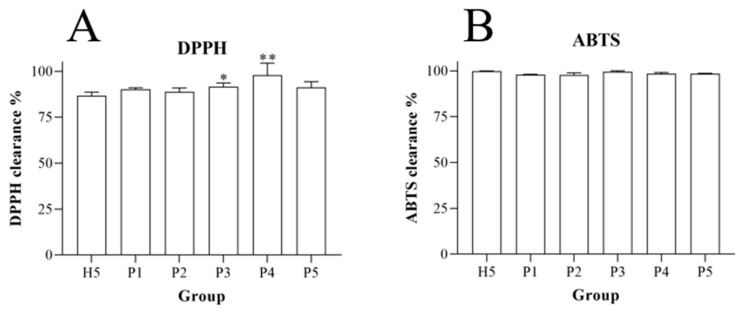
DPPH (**A**) and ABTS (**B**) antioxidant activity of Piperis Herba and Piperis Kadsurae Caulis ($\bar{x}$ ± s, n = 3), Annotations: Compare with H5, * *p* < 0.05, ** *p* < 0.005. Note: H5 is three batches of Piperis Kadsurae Caulis from Hubei Enshi. P1–P5 are individuals of three batches of Piperis Herba from different origins.

Based on the ability to differentiate between Piperis Herba and Piperis Kadsurae Caulis in terms of compounds, the antioxidant activities of Piperis Herba and Piperis Kadsurae Caulis collected from multiple origins were compared. The DPPH radical scavenging assay showed that both Piperis Herba and Piperis Kadsurae Caulis possessed significant antioxidant activities. A comparison of the antioxidant results of 15 batches of Piperis Herba and Piperis Kadsurae Caulis showed that Piperis Kadsurae Caulis from Hubei Enshi (H5) had more significant antioxidant activity among the collected Piperis Kadsurae Caulis of five origins, and its activity was compared with that of Piperis Herba of various origins (P1–P5), which was found to have a wider range of antioxidant activity than that of Piperis Kadsurae Caulis. The ABTS free radical scavenging assay again verified the significant antioxidant effects of both Piperis Herba and Piperis Kadsurae Caulis, and a comparison of their ABTS free radical scavenging rates revealed similar antioxidant activities. Although many of the compounds are common to both Piperis Herba and Piperis Kadsurae Caulis, the relative content of the compounds is quite different, and the more abundant amide alkaloids in Piperis Herba may be the basis for their more significant antioxidant activity. The comparisons of the DPPH and ABTS radical scavenging assays of Piperis Herba and Piperis Kadsurae Caulis are shown in Figure 9.

## 3. Discussion

The present study provides a better way to differentiate between Piperis Herba and Piperis Kadsurae Caulis. In the course of this study, both Piperis Herba and Piperis Kadsurae Caulis were characterized by aromatic odor and pungent taste, with Piperis Herba being more pungent, which was associated with the relatively higher content of amide alkaloids in Piperis Herba. After screening out the different compounds, we would have liked to explore the amide alkaloids plant metabolic pathway through plant metabolomics, but the metabolic pathway of this kind of compound was only slightly retrieved, and this study could not be carried out well. Considering that these are different herbal medicines, metabolomics is less used in comparisons of different drugs, so the evaluation of antioxidant activity was supplemented for preliminary validation. The limitation of this study is that there are fewer sources of origin of Piperis Herba and Piperis Kadsurae Caulis; therefore, in the subsequent study, we will collect Piperis Herba from more origins, further compare Piperis Herba and Piperis Kadsurae Caulis in analgesic and anti-inflammatory activities, and identify Piperis Herba at multiple levels through DNA molecular identification techniques while combining gene transcriptomics, so as to standardize the medicinal herbs of Piperis Herba.

## 4. Materials and Methods

### 4.1. Reagents

LC-MS-grade acetonitrile was purchased from Merck KGaA (LOT: I1233229 234, Darmstadt, Germany). Deionized water was purchased from Watsons (Guangdong, China).

### 4.2. Collection and Preparation of Medicinal Herbs

In 2023, a total of 15 batches of Piperis Herba (*Piper wallichii* (Miq.) Hand.-Mazz.) and 15 batches of Piperis Kadsurae Caulis (*Piper kadsura* (Choisy) Ohwi) were collected. Piperis Herba were collected from Guangxi, Yunnan, and Sichuan, Piperis Kadsurae Caulis were collected from Guangxi, Fujian, Sichuan, and Hubei, and detailed information regarding the herbs is shown in Supplementary Material S3. The collected herbs were authenticated by Professor Hu Yang of the Nanjing University of Chinese Medicine.

In this experiment, UPLC was firstly carried out to investigate the extraction solvents (water, 50% methanol, 75% methanol, and methanol), extraction time (15, 30, and 60 min), material–liquid ratios (1:25, 1:50, 1:100, and 1:200 *w*/*v*), and the gradient of the mobile phase (acetonitrile–water, methanol–water, acetonitrile–0.1% (*v*/*v*) formic acid in water).

All collected samples were smashed into powders and passed through a 65-mesh sieve separately. About 0.2 g of powder was weighed separately and weighed precisely, and 20 mL of 75% (*v*/*v*) ethanol was added precisely, stoppered tightly, and weighed. Following ultrasonic treatment for 30 min (240 W, 40 KHZ), cooling to room temperature, adding 75% methanol to make up the weight loss, and centrifugation at 10,000× *g* for 15 min, the supernatant was taken as a backup. At the same time, an equal amount of 30 batches of sample solution was mixed to prepare the QC samples. The samples were injected and analyzed once every 10 min to determine the stability of the instrument.

### 4.3. UHPLC-MS Analysis

The detection was performed using a high-resolution liquid–mass spectrometer ZenoTOF 7600, and a column of XDB-C8 (2.1 × 100 mm, 1.8 μm) was selected for the analysis. The column was eluted with water as mobile phase A and acetonitrile as mobile phase B in the following gradient: 0~10 min, 25–38%B; 10~16 min, 38~45%B; 16~18 min, 45~55%B; 18~22 min, 55~55%B; 22~24 min, 55~70%B; 24~26 min, 70~100%B; 26~27 min, 100~25%B; 27~30 min, 25~25%B. Flow rate 0.3 mL/min, injection volume 3 μL; column temperature 40 °C.

MS conditions in positive ion mode were set as follows: ion source temperature (TEM), 550 °C; flow rate of curtain gas (CUR), 35psi; flow rate of nebulization gas (GS1) and flow rate of auxiliary gas (GS2), 55 psi; ion spray floating voltage (ISVF), 5500 V; collision energy (CE), 35 eV; declustering potential, 100 eV. Data were acquired from 80–1250 Da for each sample. The Sample Acquisition Time was 30 min.

### 4.4. Molecular Network and Compound Identification Analysis

The molecular network was used for rapid identification of the compounds of Piperis Herba and Piperis Kadsurae Caulis, and the original mass spectral files of Piperis Herba and Piperis Kadsurae Caulis were imported into the MSConvert software of ProteoWizard 3.0.23199 64-bit and converted into the format of mzML, respectively. Then, the files were uploaded to the GNPS website (https://gnps.ucsd.edu/) (accessed on 2 November 2023) via WinSCP software 6.1. Molecular network analysis was performed by setting the precursor ion mass and fragment ion to 0.02 Da and the minimum cosine value to 0.07. The rest of the parameters were set according to the default settings and submitted to the molecular network run [31]. Cytoscape 3.9 was used to process the generated molecular network, and the compounds on the network were analyzed and identified.

Peakview 1.2 was used to analyze and verify the accuracy of the identification results using the GNPS molecular network, combined with secondary fragments from the literature, and to analyze the cleavage pattern of the compounds to determine the structure of the compounds. The identification of other unknown compounds was inferred based on the fragment information of MS/MS with the combination of Pubchem (https://pubchem.ncbi.nlm.nih.gov/) (accessed on 10 November 2023), CNKI (https://kns.cnki.net/) (accessed on 11 November 2023), SciFinder (https://scifinder.cas.org/) (accessed on 10 November 2023), and the related literature.

### 4.5. Analysis of the Differential Compounds in Piperis Herba and Piperis Kadsurae Caulis

The mass spectrometry files of 30 batches of Piperis Herba and Piperis Kadsurae Caulis were imported into Markview 1.2.1. The Minimum retention time was set to 0.1 min, the Maximum retention time was set to 30 min, and the rest of the parameters were set according to the default settings of the software. Multiple batches were processed, isotope ions were removed, common peaks were analyzed, and, finally, a *t*-test was performed to select the substances with *p* < 0.05 as the differential compounds of Piperis Herba and Piperis Kadsurae Caulis. The identified differential compounds were plotted on a heatmap for visual comparison of relative contents.

### 4.6. Comparison of Antioxidant Activity between Piperis Herba and Piperis Kadsurae Caulis

Evaluation of antioxidant activity was carried out to determine DPPH and ABTS free radical scavenging rate in 15 batches of Piperis Herba and 15 batches of Piperis Kadsurae Caulis. DPPH and ABTS free radical scavenging assays were carried out based on a methodology from the literature [11,12], and a comparison was made by integrating the different origins of Piperis Herba and the origin source with the most significant antioxidant activity of the origin source of Piperis Kadsurae Caulis to achieve a differentiation between Piperis Herba and Piperis Kadsurae Caulis in terms of antioxidant activity.

## 5. Conclusions

In conclusion, it was found that Piperis Herba has a richer compound and a wider range of antioxidant activities than Piperis Kadsurae Caulis. These results help us to better compare the differences in compounds and activity between the herbs of Piperis Herba and Piperis Kadsurae Caulis and lay an experimental foundation for further promoting the standardization and clinical application of Piperis Herba.

## Figures and Tables

**Table 1:** Identification of 72 compounds in Piperis Herba (S1) and Piperis Kadsurae Caulis (H1).

NO.	t_R_ min	Molecular Formula	[M+H]^+^	Error (ppm)	MS^2^	Compound	S1	H1	Ref.
1	0.66	C_10_H_19_NO_7_	266.1236	0.8	248.1128, 230.1024, 182.0824	D-1-[(3-Carboxypropyl)amino]-1-deoxyfructose	+	+	[13]
2	0.73	C_10_H_13_NO_2_	180.1017	−1	163.0751, 145.0651, 115.0550	1-methyl-1,2,3,4-tetrahydroisoquinoline-6,7-diol	+	+	[13]
3	0.95	C_19_H_21_NO_2_	296.1643	−0.9	265.1218	(-)-Nuciferine	−	+	[13]
4	0.96	C_27_H_30_O_15_	595.1653	−0.7	433.1128, 415.1017, 313.0700	Vitexia-Glucoside	+	+	[14]
5	0.96	C_10_H_10_O_3_	179.0696	−3.7	147.0441, 119.0491	Coniferyl aldehyde	+	+	[13]
6	0.97	C_25_H_27_NO_10_	502.1699	−1.7	337.1072, 305.0804, 201.0549	2-[[(E)-3-[2-(4-hydroxy-3-methoxyphenyl)-3-(hydroxymethyl)-7-methoxy-2,3-dihydro-1-benzofuran-5-yl]prop-2-enoyl]amino]pentanedioic acid	+	+	[13]
7	0.98	C_17_H_19_NO_3_	286.1434	−1.2	237.0907, 107.0500	Coclaurine	+	+	[15]
8	0.98	C_21_H_24_O_5_	221.1898	−0.8	219.1062, 151.0781, 135.0441	Isodihydrofutoquinol B	−	+	[13]
9	1	C_27_H_30_O_14_	579.1702	−1.2	433.1124	Vitexin-2-O-rhamnoside	+	−	[16]
10	1.06	C_19_H_23_NO_4_	330.1695	−1.5	192.1016, 137.0592	Reticuline	+	+	[17]
11	1.1	C_20_H_23_NO_4_	342.1697	−0.8	311.1264, 265.0864	Isocorydine	+	+	[13]
12	1.12	C_28_H_32_O_15_	609.1813	−0.1	447.1292, 429.1188, 327.0854	Spinosin	+	−	[18]
13	1.16	C_21_H_20_O_10_	433.1127	−0.5	313.0710, 283.0604	Vitexin	+	−	[14]
14	2.43	C_11_H_16_O_3_	197.1171	−0.8	179.1061, 133.1008, 105.0696	Loliolid	+	+	[13]
15	2.85	C_17_H_17_NO_3_	284.1281	−0.2	284.1298, 147.0485, 121.0656	Paprazine	+	+	[19]
16	4.73	C_38_H_40_N_2_O_10_	685.2752	−0.6	548.1907, 520.1955, 351.0863	(1R,2S)-7-hydroxy-1-(4-hydroxy-3,5-dimethoxyphenyl)-2-N,3-N-bis[2-(4-hydroxyphenyl)ethyl]-6,8-dimethoxy-1,2-dihydronaphthalene-2,3-dicarboxamide	+	+	[13]
17	5.15	C_18_H_20_N_2_O_2_	297.1596	−0.5	176.1158, 175.1251, 105.0421	N-(4-benzamidobutyl)benzamide	+	−	[13]
18	5.51	C_14_H_15_NO_3_	246.1125	0	175.0384, 145.0281, 98.0597	1-(3-(1,3-Benzodioxol-5-yl)-1-oxo-2-propenyl)pyrrolidine	+	+	[13]
19	6.21	C_14_H_15_NO_3_	246.1125	0	175.0380, 145.0276, 117.0337	(2E,4E)-5-(1,3-benzodioxol-5-yl)-N,N-dimethylpenta-2,4-dienamide	+	+	[13]
20	7.22	C_16_H_11_NO_3_	266.0807	−1.6	266.0811, 251.0577, 195.0687	Piperolactam A	+	+	[20]
21	7.29	C_14_H_17_NO_2_	232.1331	−0.5	232.1361, 161.0624, 133.0646	Piperlotine A	+	+	[21]
22	8.38	C_17_H_13_NO_4_	296.0916	−0.4	281.0681, 263.0570, 207.0673	Piperolactam D	+	+	[19]
23	9.5	C_15_H_17_NO_3_	260.1281	0	175.0482, 145.0375, 86.0974	Ilepcimide	+	+	[13]
24	9.53	C_16_H_17_NO_3_	272.1276	−0.9	201.0542, 135.0448, 86.0947	Piperyline	+	+	[6]
25	11.78	C_19_H_25_NO_4_	332.1855	−0.5	135.0436, 86.0962	(2E,4E)-1-piperidin-1-yl-5-(2,3,4-trimethoxyphenyl)penta-2,4-dien-1-one	+	+	[13]
26	12.14	C_23_H_31_NO_4_	386.2323	−0.8	225.1273, 135.0440, 86.0990	(2,3-dimethoxyphenyl)-[1-[2-(4-methoxyphenyl)ethyl]piperidin-4-yl]methanol	+	−	[13]
27	12.18	C_16_H_19_NO_3_	274.1436	−0.7	274.1435, 201.0544,	Piperlonguminine	+	+	[6]
28	12.62	C_16_H_35_NO_2_	274.2738	−0.8	256.2624, 106.0862	Lauryldiethanolamine	+	+	[13]
29	13.03	C_17_H_21_NO_3_	288.1592	−0.8	135.0437	Piperanine	+	+	[6]
30	13.16	C_17_H_19_NO_3_	286.1434	−0.9	201.057, 86.0963	Piperine	+	+	[6]
31	13.27	C_18_H_39_NO_3_	318.3001	−0.7	318.3051, 300.2885	Phytosphingosine	+	+	[13]
32	13.76	C_18_H_21_NO_3_	300.1595	0.1	161.0594, 131.0493, 103.0557	(2E,4E)-7-(1,3-benzodioxol-5-yl)-1-pyrrolidin-1-ylhepta-2,4-dien-1-one	+	+	[13]
33	16.18	C_16_H_14_O_4_	271.0962	−0.9	121.0232	(E)-1-(4-hydroxy-2-methoxyphenyl)-3-(4-hydroxyphenyl)prop-2-en-1-one	−	+	[13]
34	16.45	C_18_H_23_NO_3_	302.1750	−0.4	161.0682, 131.0579, 103.0621	Futoamide	+	+	[22]
35	17.12	C_19_H_21_NO_3_	312.1592	−0.7	169.0643, 131.0495, 86.0985	Piperettine	+	+	[13]
36	17.33	C_19_H_23_NO_3_	314.1749	−0.6	161.0676, 131.0575, 86.1027	Pipersintenamide	+	+	[23]
37	17.83	C_18_H_25_NO_3_	304.1906	−0.3	203.1064, 135.0449, 123.0438	Pipercallosidine	+	+	[13]
38	17.93	C_14_H_23_NO	222.1851	−0.9	151.1115, 81.0343	Spilanthol	+	+	[23]
39	18.65	C_19_H_25_NO_3_	316.1907	−0.1	135.0469, 86.0971	Piperolein A	+	+	[6]
40	18.85	C_20_H_25_NO_3_	328.1907	−0.1	229.1249, 135.0484	Retrofractamide A	+	+	[6]
41	18.87	C_22_H_28_O_5_	373.2010	0.1	151.0843, 139.0774	Galgravin	−	+	[13]
42	19.08	C_15_H_22_O	219.1742	−0.8	163.1135, 93.0702, 81.0714	Nootkatone	+	+	[24]
43	19.12	C_18_H_32_O_2_	281.2472	−1	133.1006, 105.0690, 91.0538	Linoleic acid	+	−	[13]
44	19.39	C_18_H_39_NO_2_	302.3051	−0.8	284.2922, 106.0855, 88.0759	Tetradecyldiethanolamine	+	+	[13]
45	19.55	C_14_H_25_NO	224.2007	−0.9	151.1123, 133.1007, 81.0534	Pellitorine	+	+	[6]
46	20.13	C_20_H_27_NO_3_	330.2064	0.1	229.1235, 135.0450	Retrofractamide C	+	+	[22]
47	20.17	C_21_H_25_NO_3_	340.1905	−0.6	227.1070, 131.0490, 86.0962	Dehydropipernonaline	+	+	[22]
48	20.55	C_20_H_29_NO_3_	332.2219	−0.4	135.0443	(E)-9-(1,3-benzodioxol-5-yl)-N-(2-methylpropyl)non-8-enamide	+	+	[13]
49	20.7	C_32_H_30_N_2_O_4_	507.2275	−0.6	256.1321, 238.1221, 105.0331	Asperphenamate	+	+	[13]
50	21.09	C_21_H_27_NO_3_	342.2062	−0.4	229.1221, 135.0459, 86.0969	Pipernonaline	+	+	[22]
51	21.31	C_22_H_27_NO_3_	354.2061	−0.7	135.0438, 131.0485	(2E,4E,10E)-11-(1,3-benzodioxol-5-yl)-1-pyrrolidin-1-ylundeca-2,4,10-trien-1-one	+	+	[13]
52	21.69	C_15_H_27_NO	238.2164	−0.8	168.1384, 81.0697	(2E,4E)-N-Isobutylundeca-2,4-dienamide	+	+	[13]
53	22.34	C_21_H_29_NO_3_	344.2220	−0.1	135.0468, 112.0754, 86.0969	Piperolein B	+	+	[6]
54	22.66	C_22_H_29_NO_3_	356.2220	−0.2	135.0430, 131.0481	Retrofractamide B	+	+	[6]
55	22.72	C_16_H_27_NO	250.2164	−0.7	124.0754, 98.0599	(2E,4E)-1-(1-Pyrrolidinyl)-2,4-dodecadien-1-one	+	+	[25]
56	23.14	C_22_H_29_NO_3_	356.2220	−0.2	135.0450, 131.0489, 98.0606	(4E,10E)-11-(1,3-benzodioxol-5-yl)-1-pyrrolidin-1-ylundeca-4,10-dien-1-one	+	+	[13]
57	23.62	C_29_H_39_N_3_O_2_	462.3117	0.4	406.2469, 338.1844, 198.1271	Echinulin	+	+	[13]
58	24.18	C_22_H_31_NO_3_	358.2377	0.2	161.0597, 135.0449	Piperchabamide D	+	+	[26]
59	24.27	C_23_H_29_NO_3_	368.2216	−1.2	225.1358, 135.0444, 86.0968	Piperundecalidine	+	+	[22]
60	24.38	C_16_H_29_NO	252.2321	-0.3	196.1694, 179.1435, 95.0851	(2E,4E)-N-(2-methylpropyl)dodeca-2,4-dienamide	+	+	[6]
61	24.98	C_23_H_31_NO_3_	370.2376	−0.3	135.0437, 131.0494, 86.0962	Piperchabamide B	+	−	[6]
62	25.02	C_20_H_42_O_5_	363.3104	−0.2	195.1213, 133.0850	3,6,9,12-Tetraoxatetracosan-1-ol	+	+	[13]
63	25.26	C_17_H_29_NO	264.2320	−0.8	112.0754, 86.0963	(2E,4E)-N-dodecadienoylpiperidine	+	−	[27]
64	25.44	C_17_H_31_NO	266.2476	−1	112.0749, 95.0849	(2E,4E)-N-ethyl-3,7,11-trimethyldodeca-2,4-dienamide	+	+	[13]
65	25.5	C_24_H_33_NO_3_	384.2532	−0.3	283.1693, 135.0469, 86.0987	Guineensine	+	+	[6]
66	25.5	C_23_H_33_NO_3_	372.2530	−0.9	135.0438, 86.0964	(2E,11E)-12-(1,3-benzodioxol-5-yl)-N-(2-methylpropyl)dodeca-2,11-dienamide	+	−	[6]
67	25.79	C_19_H_32_O_2_	293.2474	−0.5	243.2090, 137.1314	Methyl alpha-eleostearate	+	+	[6]
68	25.83	C_24_H_35_NO_3_	386.2686	−0.9	313.1802, 135.0439	(2E,4E)-5-(1,3-benzodioxol-5-yl)-N,N-dihexylpenta-2,4-dienamide	+	−	[6]
69	25.93	C_25_H_33_NO_3_	396.2530	−0.9	135.0438, 131.0485, 86.0960	Piperchabamide C	+	−	[6]
70	25.99	C_16_H_33_NO	256.2632	−1.3	102.0910, 88.0756	Palmitamide	+	+	[28]
71	26.33	C_26_H_37_NO_3_	412.2845	−0.3	339.1948, 135.0435, 86.0960	Brachystamide B	+	+	[6]
72	26.33	C_18_H_35_NO	282.2789	−1	265.2513, 247.2419, 149.1321	Oleamide	+	+	[29]

Note: “+” indicates that the substance has been identified. “−” indicates that the substance has not been identified.

## Data Availability

The data presented in this study are available within the article.

## Supplementary Materials

The following supporting information can be downloaded at: https://www.mdpi.com/article/10.3390/molecules29020439/s1, Supplementary Material S1; Supplementary Material S2; Supplementary Material S3.
