# Supplementary material for: Rapid Analysis of Compounds from Piperis Herba and Piperis Kadsurae Caulis and Their Differences Using High-Resolution Liquid–Mass Spectrometry and Molecular Network Binding Antioxidant Activity
Source: Molecules. 2024 Jan 16;29(2):439. doi: 10.3390/molecules29020439 (PMC10821392; doi:10.3390/molecules29020439)
Supplement: Supplementary file 1 [file molecules-29-00439-s001.zip › supplementary material S1.pdf]

In order to better detect the compounds, we optimized the conditions by taking the representative of Piperis Herba, and examined the extraction solvent, extraction time, feed/liquid ratio and mobile phase, respectively. After comparison, we chose 75% methanol as the extraction solvent for 30 min with a feed/liquid ratio of 1:100, and acetonitrile and water as the mobile phases. The results of mass spectrometry showed that the conditions had good applicability to both Piperis Herba and Piperis Kadsurae Caulis, as follows:

Extraction Solvents

Water

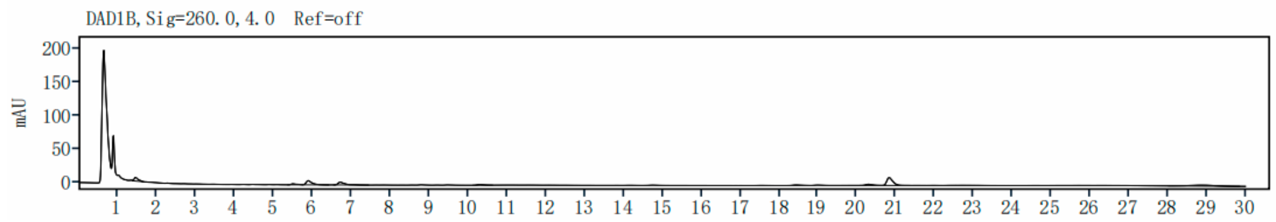

t/min

50% methanol

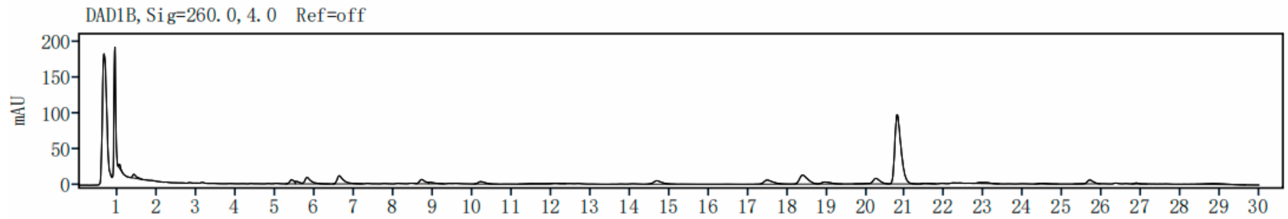

t/min

75% methanol

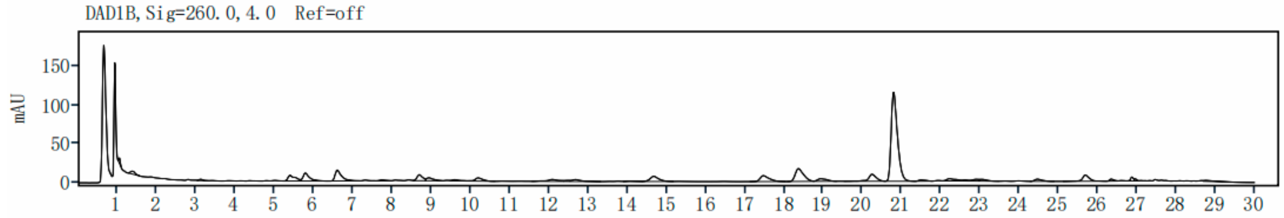

t/min

methanol

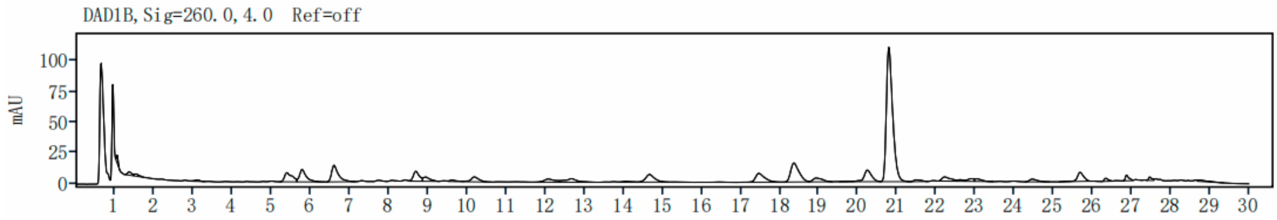

t/min

Extraction Time

15min

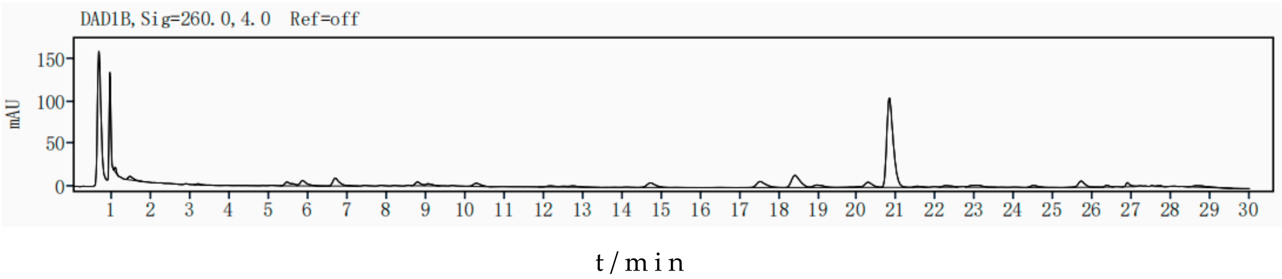

30min

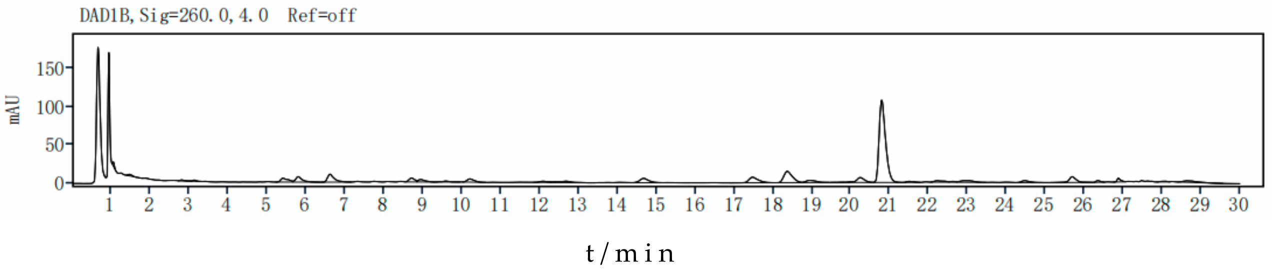

60min

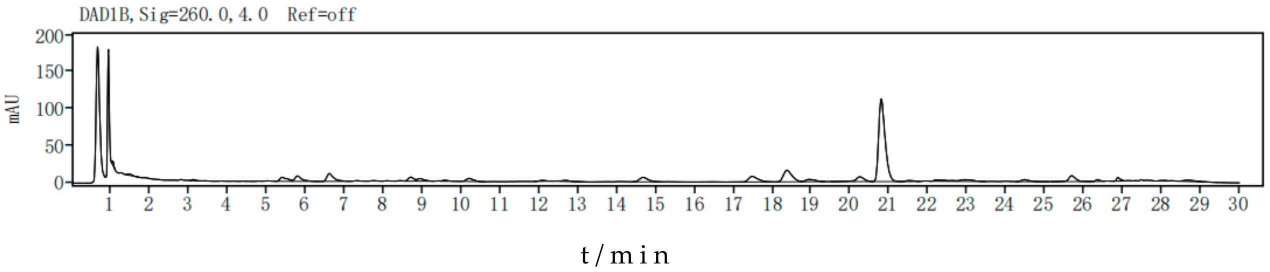

Material-liquid Ratios

(1:25 w/v)

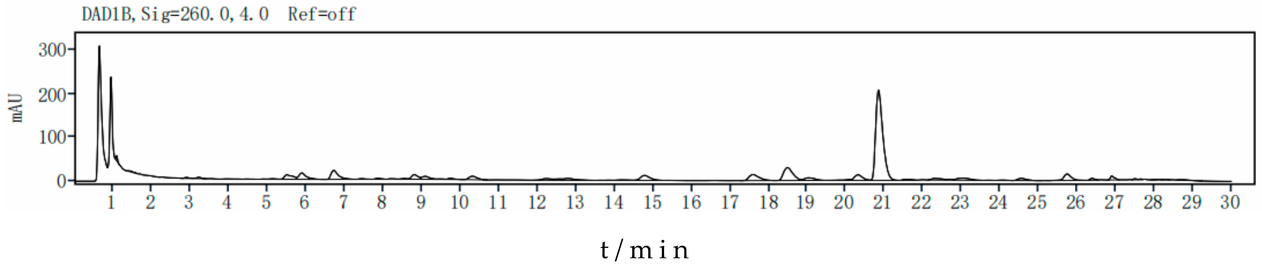

(1:50 w/v)

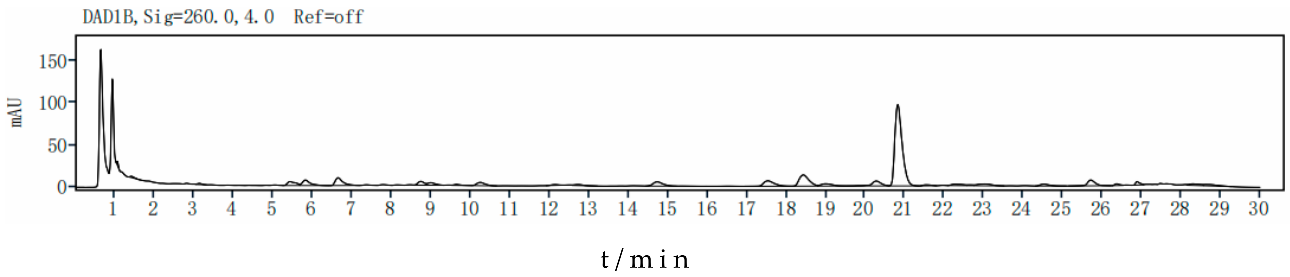

(1:100 w/v)

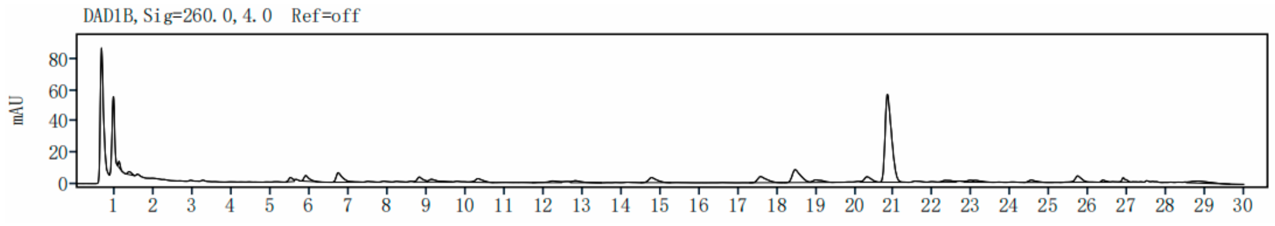

t/min

(1:200 w/v)

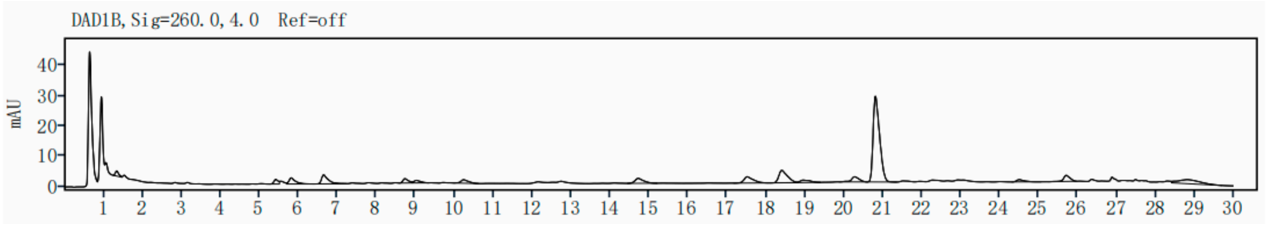

t/min

Gradient of the Mobile Phase

Acetonitrile-water

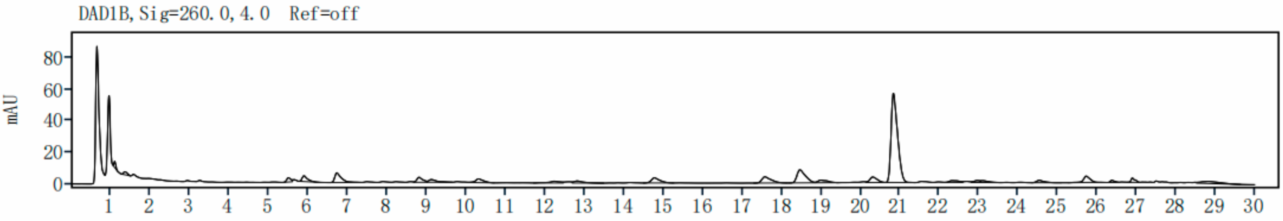

t/min

Methanol-water

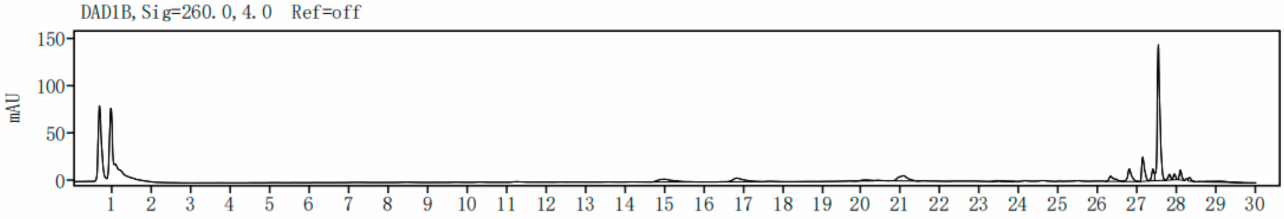

t/min

Acetonitrile-0.1% (v/v) formic acid in water

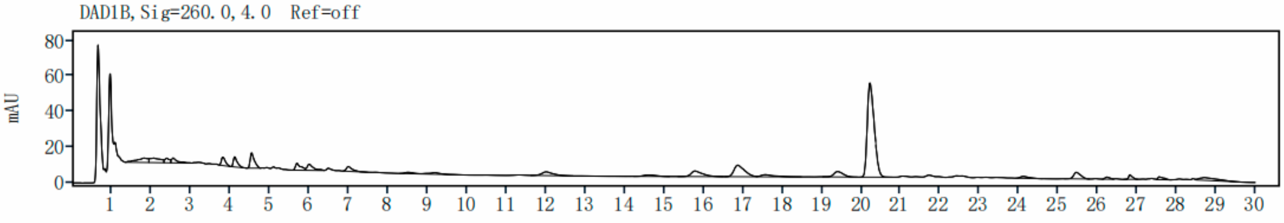

t/min
