# Supplementary material for: Rapid Analysis of Compounds from Piperis Herba and Piperis Kadsurae Caulis and Their Differences Using High-Resolution Liquid–Mass Spectrometry and Molecular Network Binding Antioxidant Activity
Source: Molecules. 2024 Jan 16;29(2):439. doi: 10.3390/molecules29020439 (PMC10821392; doi:10.3390/molecules29020439)

1. D-1-[(3-Carboxypropyl)amino]-1-deoxyfructose

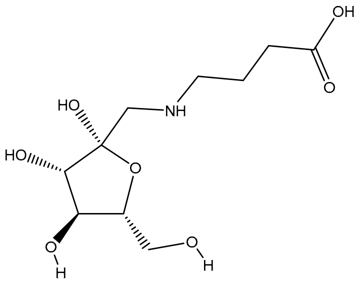

2. 1-methyl-1,2,3,4-tetrahydroisoquinoline-6,7-diol

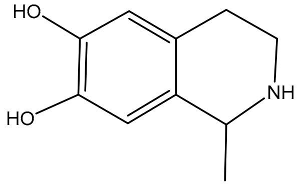

3. (-)-Nuciferine

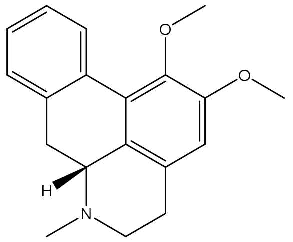

4. Vitexia-Glucoside

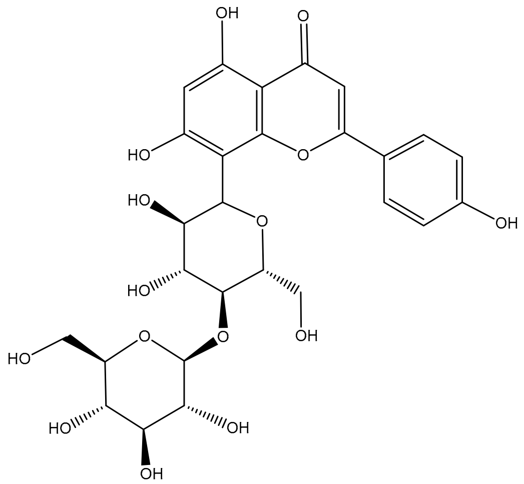

5. Coniferyl aldehyde

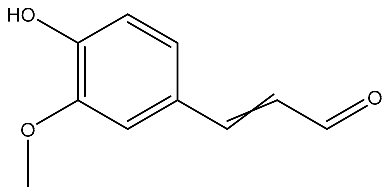

6. 2-[[(E)-3-[2-(4-hydroxy-3-methoxyphenyl)-3-(hydroxymethyl)-7-methoxy-2,3-dihydro-1-benzofuran-5-yl]prop-2-enoyl]amino]pentanedioic acid

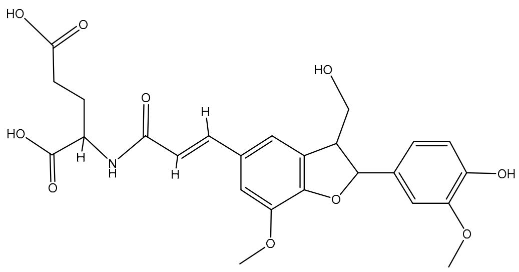

7. Coclaurine

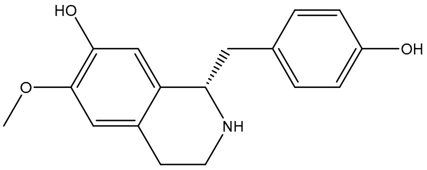

8. Isodihydrofutoquinol B

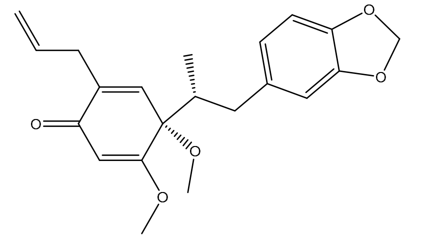

9. Vitexin-2-O-rhamnoside

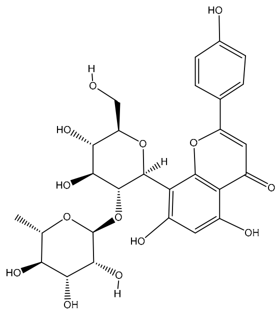

10. Reticuline

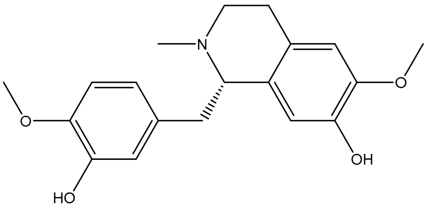

11. Isocorydine

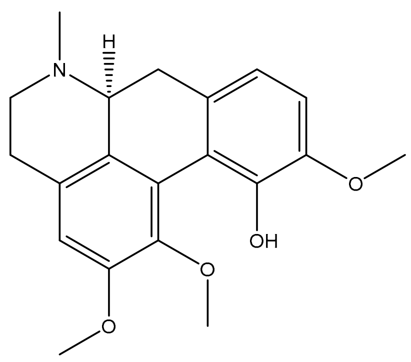

12. Spinosin

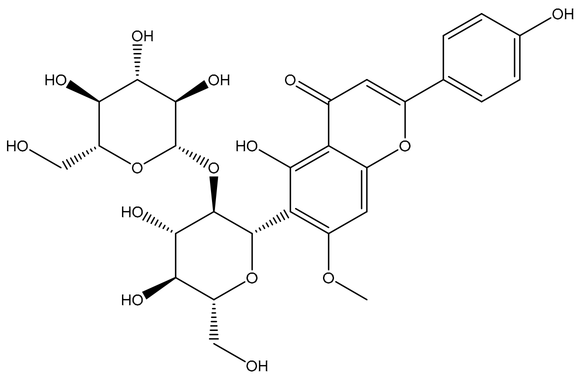

13. Vitexin

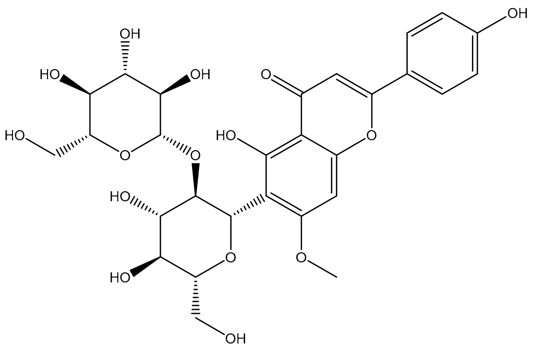

14. Loliolid

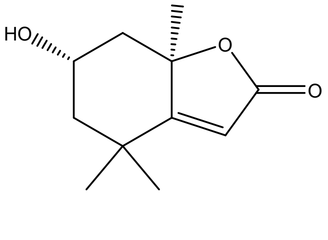

15. Paprazine

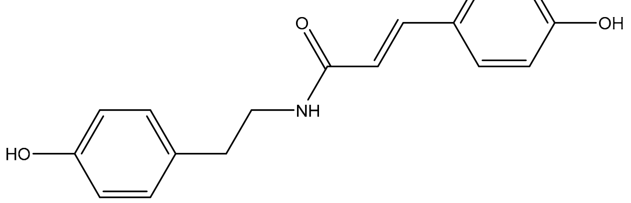

16. (1R,2S)-7-hydroxy-1-(4-hydroxy-3,5-dimethoxyphenyl)-2-N,3-N-bis[2-(4-hydroxyphenyl)ethyl]-6,8-dimethoxy-1,2-dihydronaphthalene-2,3-dicarboxamide

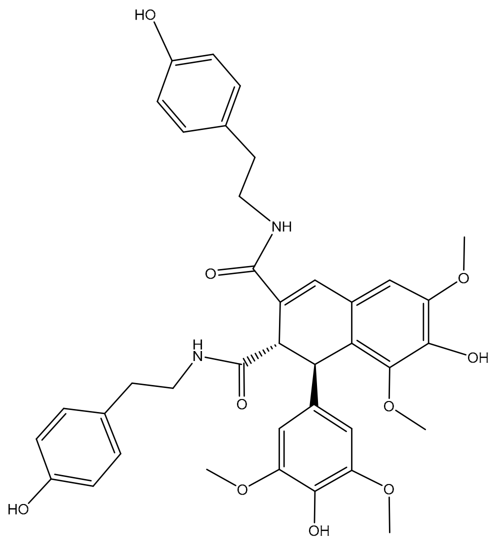

17. N-(4-benzamidobutyl)benzamide

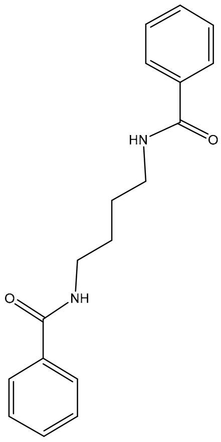

18. (2E,4E)-5-(1,3-benzodioxol-5-yl)-N,N-dimethylpenta-2,4-dienamide

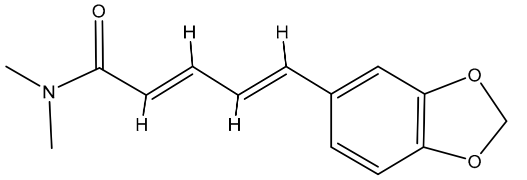

19. 1-(3-(1,3-Benzodioxol-5-yl)-1-oxo-2-propenyl)pyrrolidine

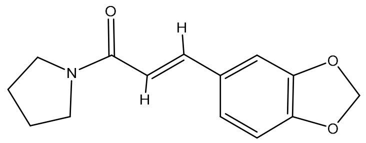

20. Piperolactam A

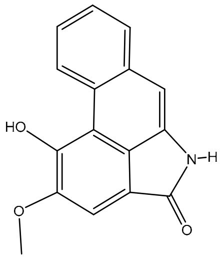

21. Piperlotine A

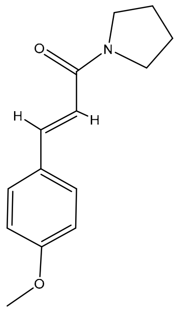

22. Piperolactam D

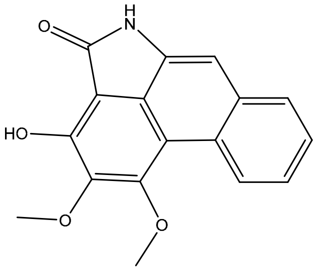

23. Ilepcimide

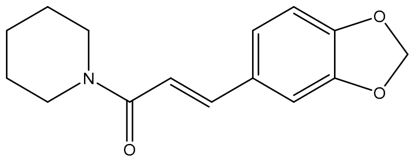

24. Piperyline

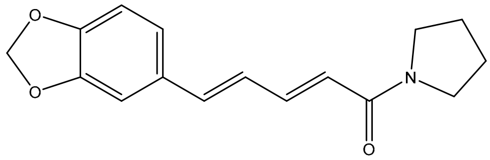

25. (2E,4E)-1-piperidin-1-yl-5-(2,3,4-trimethoxyphenyl)penta-2,4-dien-1-one

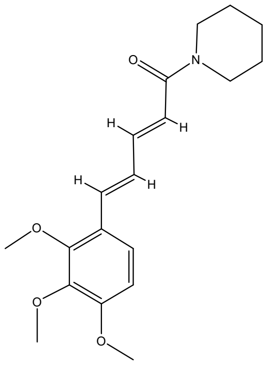

26. (2,3-dimethoxyphenyl)-[1-[2-(4-methoxyphenyl)ethyl]piperidin-4-yl]methanol

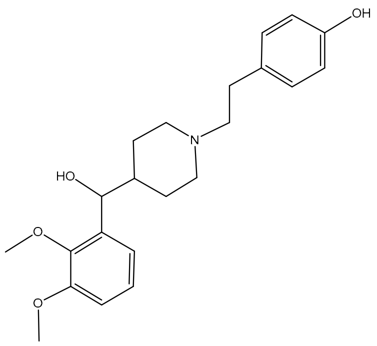

27. Piperlonguminine

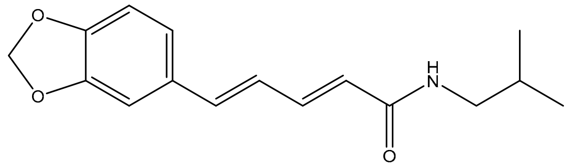

28. Lauryldiethanolamine

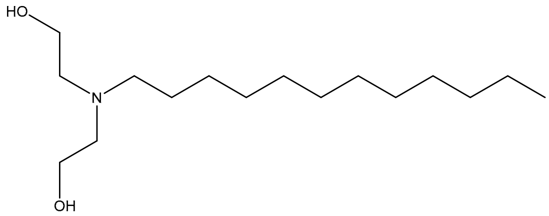

29. Piperanine

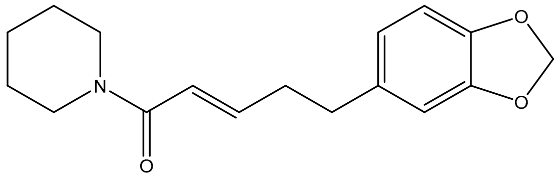

30. Piperine

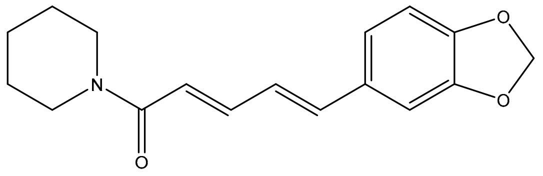

31. Phytosphingosine

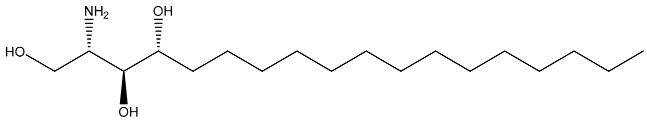

32. (2E,4E)-7-(1,3-benzodioxol-5-yl)-1-pyrrolidin-1-ylhepta-2,4-dien-1-one

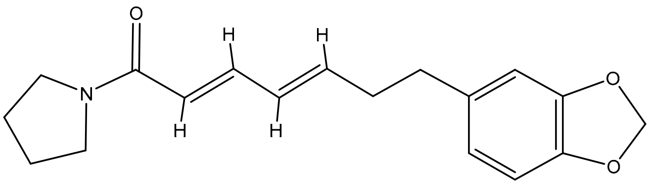

33. (E)-1-(4-hydroxy-2-methoxyphenyl)-3-(4-hydroxyphenyl)prop-2-en-1-one

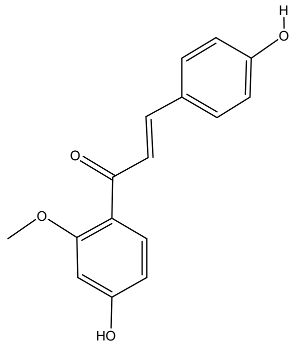

34. Futoamide

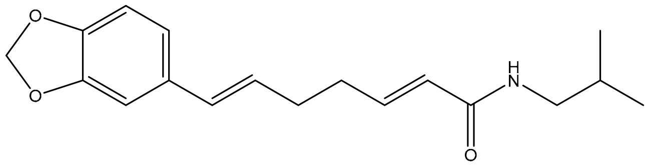

35. piperettine

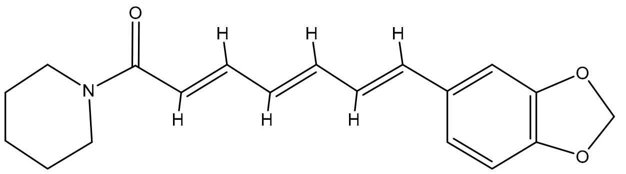

36. Pipersintenamide

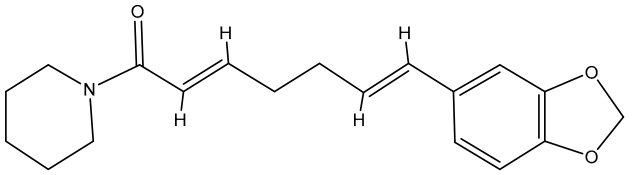

37. Pipercallosidine

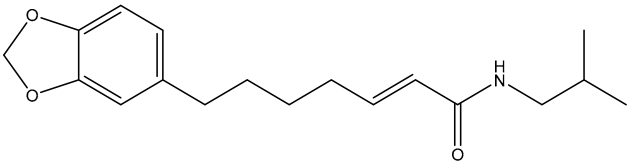

38. Spilanthol

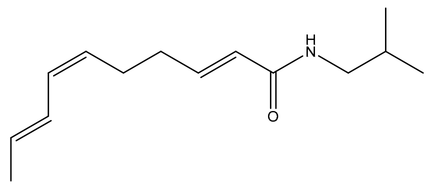

39. Piperolein A

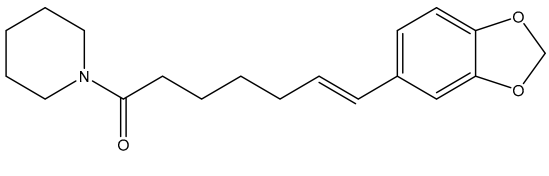

40. Retrofractamide A

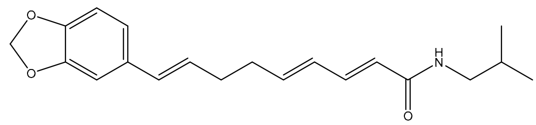

41. Galgravin

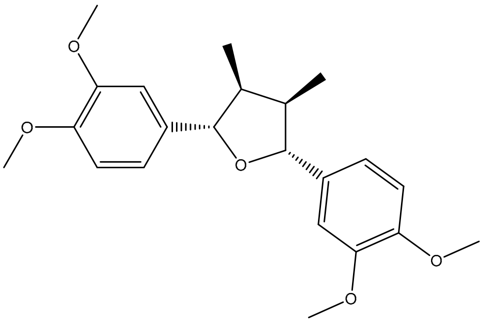

42. Nootkatone

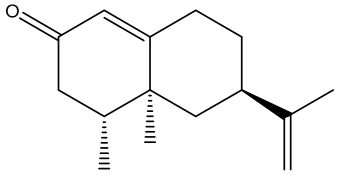

43. Linoleic acid

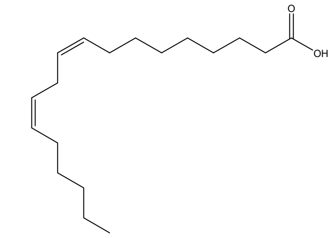

44. Tetradecyldiethanolamine

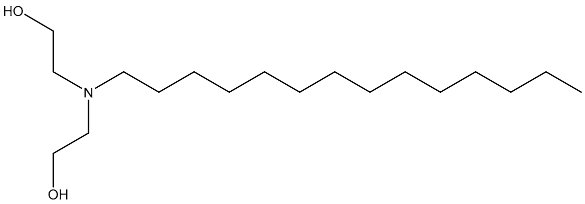

45. Pellitorine

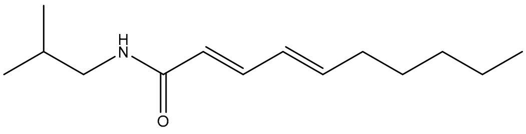

46. Retrofractamide C

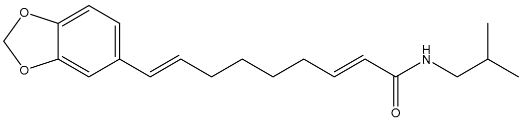

47. Dehydropipernonaline

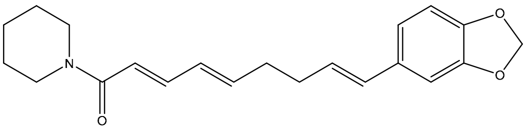

48. (E)-9-(1,3-benzodioxol-5-yl)-N-(2-methylpropyl)non-8-enamide

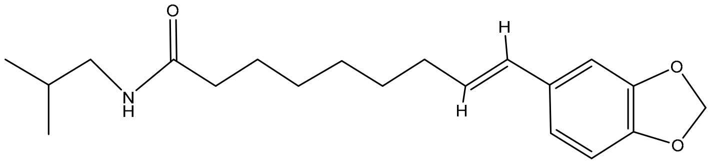

49. Asperphenamate

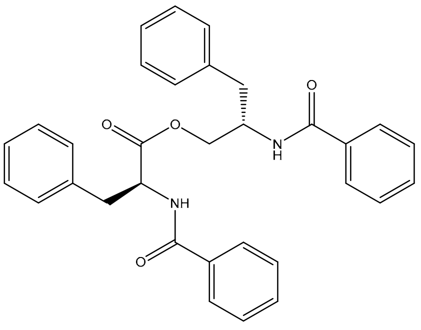

50. Pipernonaline

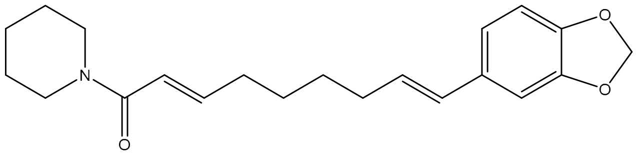

51. (2E,4E,10E)-11-(1,3-benzodioxol-5-yl)-1-pyrrolidin-1-ylundeca-2,4,10-trien-1-one

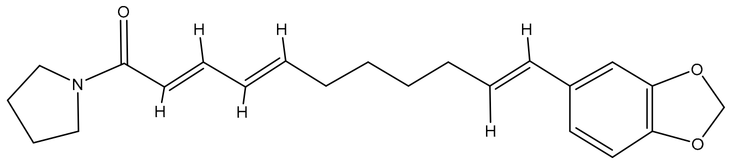

52. (2E,4E)-N-Isobutylundeca-2,4-dienamide

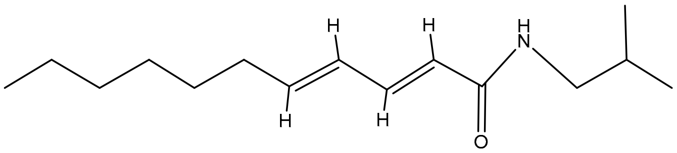

53. Piperolein B

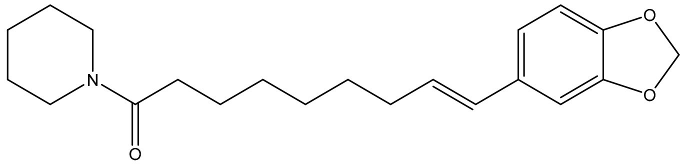

54. Retrofractamide B

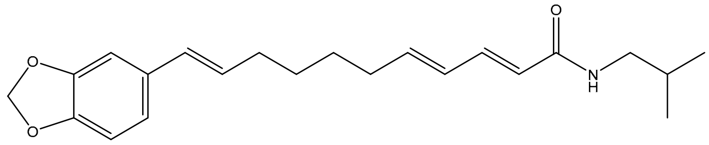

55. (2E,4E)-1-(1-Pyrrolidinyl)-2,4-dodecadien-1-one

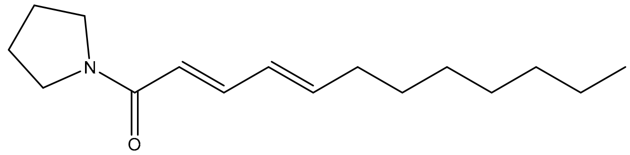

56. (4E,10E)-11-(1,3-benzodioxol-5-yl)-1-pyrrolidin-1-ylundeca-4,10-dien-1-one

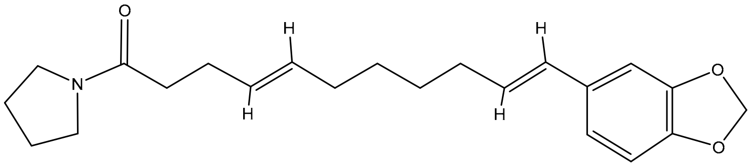

57. echinulin

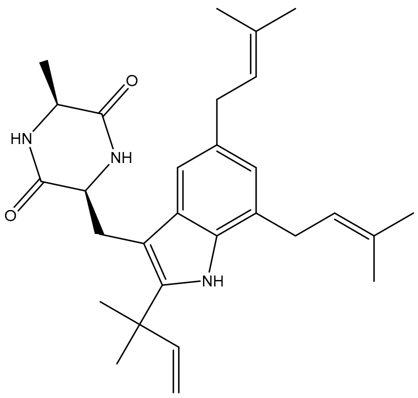

58. Piperchabamide D

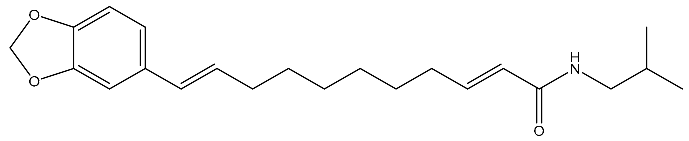

59. Piperundecalidine

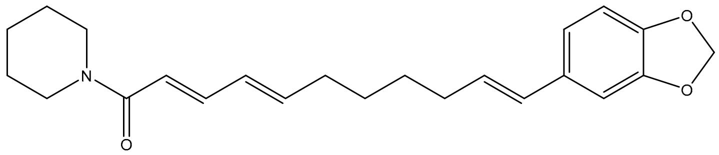

60. (2E,4E)-N-(2-methylpropyl)dodeca-2,4-dienamide

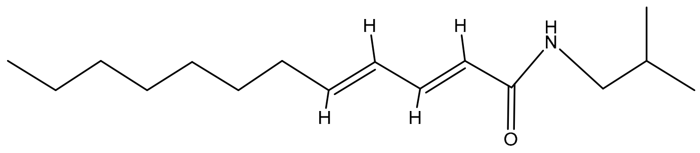

61. Piperchabamide B

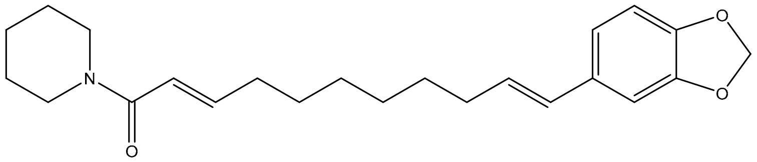

62. 3,6,9,12-Tetraoxatetracosan-1-ol

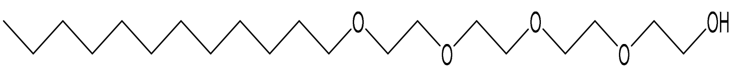

63. (2E,4E)-N-dodecadienoylpiperidine

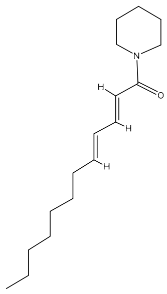

64. (2E,4E)-N-ethyl-3,7,11-trimethyldodeca-2,4-dienamide

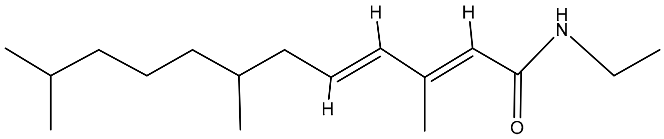

65. Guineensine

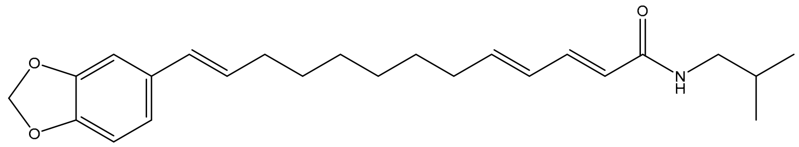

66. (2E,11E)-12-(1,3-benzodioxol-5-yl)-N-(2-methylpropyl)dodeca-2,11-dienamide

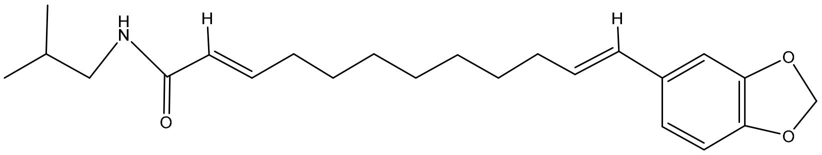

67. Methyl alpha-eleostearate

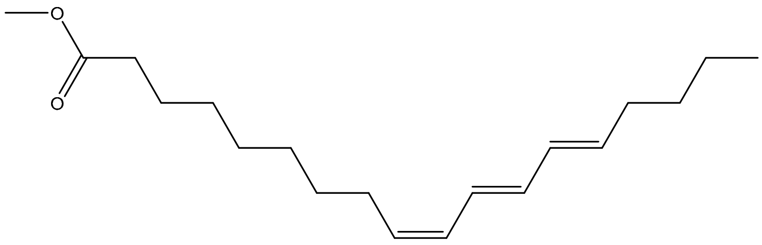

68. (2E,4E)-5-(1,3-benzodioxol-5-yl)-N,N-dihexylpenta-2,4-dienamide

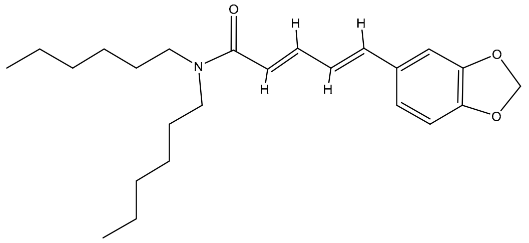

69. Piperchabamide C

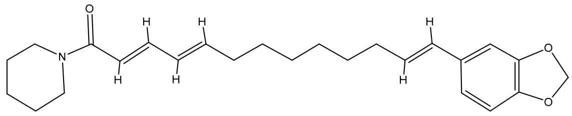

70. Palmitamide

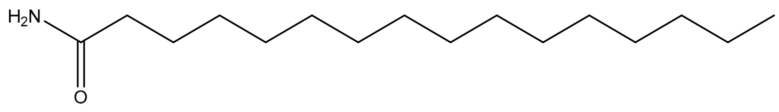

71. Brachystamide B

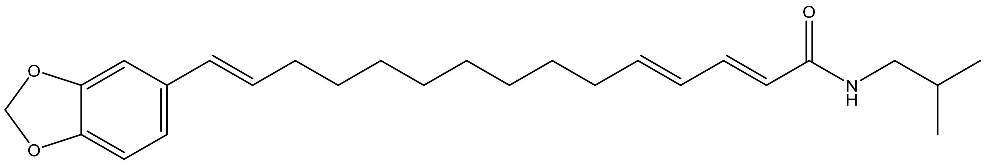

72. Oleamide

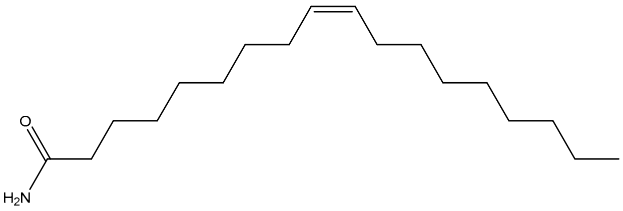

Supplement: Supplementary file 1 [file molecules-29-00439-s001.zip › supplementary material S2.pdf]
