# Supplementary material for: Rapid Analysis of Compounds from Piperis Herba and Piperis Kadsurae Caulis and Their Differences Using High-Resolution Liquid–Mass Spectrometry and Molecular Network Binding Antioxidant Activity
Source: Molecules. 2024 Jan 16;29(2):439. doi: 10.3390/molecules29020439 (PMC10821392; doi:10.3390/molecules29020439)
Supplement: Supplementary file 1 [file molecules-29-00439-s001.zip › supplementary material S3.pdf]

Table S1 Herbal Information Sheet for Piperis Herba

NO.	Origins of Piperis Herba	Group	Longitude and Latitude	Relative Humidity	Mean Temperature
S1	Guangxi Guilin	P1	N:110°11'4.624" E:25°14'37.179"	78%	20.2℃
S2	Guangxi Guilin				
S3	Guangxi Guilin				
S4	Guangxi Liuzhou	P2	N:109°26'5.482" E:24°20'6.912"	77%	21.9℃
S5	Guangxi Liuzhou				
S6	Guangxi Liuzhou				
S7	Guangxi Baise	P3	N:106°37'27.999" E:23°54'30.900"	78%	20.8℃
S8	Guangxi Baise				
S9	Guangxi Baise				
S10	Yunnan Dali	P4	N:100°18'38.890" E:25°41'11.643"	29%	20.0℃
S11	Yunnan Dali				
S12	Yunnan Dali				
S13	Guangxi Yulin	P5	N: 110°11'16.880" E: 22°39'43.557"	81%	21.2℃
S14	Guangxi Yulin				
S15	Guangxi Yulin				

Table S2 Herbal Information Sheet for Piperis Kadsurae Caulis

NO.	Origins of Piper Wallichii	Group	Longitude and Latitude	Relative Humidity	Mean Temperature
H1	Sichuan Yunlian	H1	N: 104°30'45.001"<br>E: 28°9'51.517"	48%	18.0℃
H2	Sichuan Yunlian				
H3	Sichuan Yunlian				
H4	Fujian Nanjing	H2	N: 117°21'52.021"<br>E: 24°31'16.483"	43%	20.6℃
H5	Fujian Nanjing				
H6	Fujian Nanjing				
H7	Sichuan Guangyuan	H3	N: 105°51'3.062"<br>E: 32°26'42.546"	49%	18.3℃
H8	Sichuan Guangyuan				
H9	Sichuan Guangyuan				
H10	Sichuan Bazhong	H4	N: 106°45'4.141"<br>E: 31°52'28.142"	51%	17.0℃
H11	Sichuan Bazhong				
H12	Sichuan Bazhong				
H13	Hubei Enshi	H5	N: 109°29'18.319"<br>E: 30°18'14.958"	38%	19.7℃
H14	Hubei Enshi				
H15	Hubei Enshi				

Both herbs we collected were from 1-year-old plants, harvested in summer and autumn at the end of flowering and fruiting.
